# Supplementary material for: Multigene Phylogeny Reveals Endophytic Xylariales Novelties from Dendrobium Species from Southwestern China and Northern Thailand
Source: J Fungi (Basel). 2022 Feb 28;8(3):248. doi: 10.3390/jof8030248 (PMC8955275; doi:10.3390/jof8030248)
Supplement: Supplementary file 1 [file jof-08-00248-s001.zip › jof-1587243-supplementary.pdf]

Supplementary Tables

Table S1. Strains used for phylogenetic tree construction in Apiosporaceae (*Arthrinium* and *Nigrospora*).

Species	Strain	GenBank Accession No.				Reference
		ITS	LSU	TUB2	TEF1-α	
Apiospora aquaticum	**MFLU 18-1628**	MK828608	MK835806	–	–	Luo et al. 2019
Apiospora arundinis	CBS 124788	KF144885	KF144929	KF144975	KF145017	Crous and Groenewald 2013
	LC4951	KY494698	KY494774	KY705168	KY705097	Wang et al. 2018
Apiospora aureum	**CBS 244.83**	AB220251	KF144935	KF144981	KF145023	Crous and Groenewald 2013
Apiospora balearicum	**CBS 145129**	MK014869	MK014836	MK017975	–	Pintos et al. 2019
Apiospora bambusae	**CGMCC 3.18335**	KY494718	KY494794	KY705186	KY806204	Wang et al. 2018
	LC7107	KY494719	KY494795	KY705187	KY705117	Wang et al. 2018
Apiospora bambusicola	**MFLUCC 20-0144**	MW173030	MW173087	–	MW183262	Tang et al. 2020
Apiospora camelliae-sinensis	**CGMCC 3.18333**	KY494704	KY494780	KY705173	KY705103	Wang et al. 2018
	LC8181	KY494761	KY494837	KY705229	KY705157	Wang et al. 2018
Apiospora chinense	**CFCC 53036**	MK819291	–	MK818547	MK818545	Jiang et al. 2020
Apiospora chromolaenae	**MFLUCC 17-1505**	MT214342	MT214436	–	–	Mapook et al. 2020
Apiospora dendrobii	**MFLUCC 14-0152**	MZ463151	MZ463192			This study
Apiospora descalsii	**CBS 145130**	MK014870	MK014837	MK017976	–	Pintos et al. 2019
Apiospora dichotomanthi	**CGMCC 3.18332**	KY494697	KY494773	KY705167	KY705096	Wang et al. 2018
	LC8175	KY494755	KY494831	KY705223	KY705151	Wang et al. 2018
Apiospora esporlense	**CBS 145136**	MK014878	MK014845	MK017983	MK017954	Pintos et al. 2019

Apiospora euphorbiae	IMI 285638b	AB220241	AB220335	AB220288	–	Ogawa et al. 2005
Apiospora gaoyouense	**CFCC 52301**	MH197124	–	MH236789	MH236793	Jiang et al. 2020
Apiospora garethjonesii	KUMCC 16-0202	KY356086	KY356091	–	–	Dai et al. 2016
Apiospora guizhouense	**CGMCC3.18334**	KY494709	KY494785	KY705178	KY705108	Wang et al. 2018
	LC5318	KY494708	KY494784	KY705177	KY705107	Wang et al. 2018
Arthrinium gutiae	**CBS 135835**	KR011352	MH877577	KR011350	KR011351	Kajale et al. 2015
Apiospora hispanicum	**IMI 326877**	AB220242	AB220336	AB220289	–	Ogawa et al. 2005
Apiospora hydei	**CBS 114990**	KF144890	KF144936	KF144982	KF145024	Crous and Groenewald 2013
	LC7103	KY494715	KY494791	KY705183	KY705114	Crous and Groenewald 2013
Apiospora hyphopodii	**MFLUCC 15-003**	KR069110	KY356093	–	–	Dai et al. 2016
Apiospora hysterinum	ICMP 6889	MK014874	MK014841	MK017980	MK017951	Pintos et al. 2019
Apiospora ibericum	**CBS 145137**	MK014879	MK014846	MK017984	–	Pintos et al. 2019
Apiospora italicum	**AP221017**	MK014880	MK014847	MK017985	MK017956	Pintos et al. 2019
Apiospora jatrophae	**MMI 00052**	JQ246355	–	–	–	Sharma et al. 2012
Apiospora jiangxiense	**CGMCC 3.18381**	KY494693	KY494769	KY705163	KY705092	Wang et al. 2018
	LC4578	KY494694	KY494770	KY705164	KY705093	Wang et al. 2018
Apiospora kogelbergense	**CBS 113333**	KF144892	KF144938	KF144984	KF145026	Crous and Groenewald 2013
	CBS 113332	KF144891	KF144937	KF144983	KF145025	Crous and Groenewald 2013
Apiospora locuta-pollinis	**LC11683**	MF939595	–	MF939622	MF939616	Zhao et al. 2017
Apiospora longistromum	**MFLUCC 11-0481**	KU940141	KU863129	–	–	Dai et al. 2016
	MFLUCC 11-0479	KU940142	KU863130	–	–	Dai et al. 2016

Apiospora malaysianum	**CBS 102053**	KF144896	KF144942	KF144988	KF145030	Crous and Groenewald 2013
Apiospora marii	**CBS 497.90**	AB220252	KF144947	KF144993	KF145035	Ogawa et al. 2005
Apiospora mediterranei	**IMI 326875**	AB220243	AB220337	AB220290	–	Ogawa et al. 2005
Apiospora mytilomorphum	**DAOM 214595**	KY494685	–	–	–	Wang et al. 2018
Apiospora neogarethjonesii	**KUMCC 18-0192**	MK070897	MK070898	–	–	Dai et al. 2019
Apiospora neosubglobosum	**KUMCC 16-0203**	KY356090	KY356095	–	–	Dai et al. 2016
	JHB006	KY356089	KY356094	–	–	Dai et al. 2016
Apiospora obovatum	**CGMCC 3.18331**		–	–	–	Wang et al. 2018
	LC8177	KY494757	KY494833	KY705226	KY705154	Wang et al. 2018
Apiospora ovatum	**CBS 115042**	KF144903	KF144950	KF144995	KF145037	Crous and Groenewald 2013
Apiospora paraphaeospermum	MFLUCC 13-0644	KX822128	KX822124	–	–	Senanayakeet al. 2016
Apiospora phaeospermum	CBS 114314	KF144904	KF144951	KF144996	KF145038	Crous and Groenewald 2013
Apiospora phaeospermum	CBS 114315	KF144905	KF144952	KF144997	KF145039	Crous and Groenewald 2013
Apiospora phragmits	**CPC 18900**	KF144909	KF144956	KF145001	KF145043	Crous and Groenewald 2013
Apiospora piptatheri	**CBS 145149**	MK014893	MK014860	–	–	Pintos et al. 2019
Apiospora phyllostachium	**MFLUCC 18-1101**	MK351842	MH368077	MK291949	MK340918	Yang et al. 2019
Apiospora pseudoparenchymaticum	**LC7234**	KY494743	KY494819	KY705211	KY705139	Wang et al. 2018
	LC8173	KY494753	KY494829	KY705221	KY705149	Wang et al. 2018
Apiospora pseudosinense	**CPC 21546**	KF144910	KF144957	–	KF145044	Crous and Groenewald 2013

Apiospora pseudospegazzinii	**CBS 102052**	KF144911	KF144958	KF145002	KF145045	Crous and Groenewald 2013
Apiospora pterospermum	**CPC 20193**	KF144913	KF144960	KF145004	KF145046	Crous and Groenewald 2013
Apiospora qinlingense	**CFCC 52303**	MH197120	–	MH236791	MH236795	Jiang et al. 2020
Apiospora rasikravindrae	**NFCCI 2144**	JF326454	–	–	–	Schoch et al. 2014
	LC5449	KY494713	KY494789	KY705182	KY705112	Wang et al. 2018
Apiospora sacchari	CBS 212.30	KF144916	AB220351	KF145005	KF145047	Crous and Groenewald 2013
	CBS 301.49	KF144917	AB220352	KF145006	KF145048	Crous and Groenewald 2013
Apiospora saccharicola	CBS 334.86	AB220257	KF144967	KF145010	KF145052	Crous and Groenewald 2013
	CBS 463.83	KF144921	KF144968	KF145011	KF145053	Crous and Groenewald 2013
Apiospora serenense	**IMI 326869**	AB220250	AB220344	AB220297	–	Ogawa et al. 2005
Apiospora setostromum	**KUMCC 19-0217**	MN528012	MN528011	–	MN527357	Jiang et al. 2019
Apiospora subglobosum	**MFLUCC 11-0397**	KR069112	KR069113	–	–	Senanayake et al. 2015
Apiospora subroseum	**CGMCC3.18337**	KY494752	KY494828	KY705220	KY705148	Wang et al. 2018
	LC7291	KY494751	KY494827	KY705219	KY705147	Wang et al. 2018
Apiospora thailandicum	**MFLUCC 15-0202**	KU940145	KU863133	–	–	Dai et al. 2016
	LC5630	KY494714	KY494790	KY806200	KY705113	Wang et al. 2018
Apiospora vietnamensis	**IMI 99670**	KX986096	KX986111	KY019466	–	Wang et al. 2018
Apiospora xenocordella	**CBS 478.86**	KF144925	–	KF145013	KF145055	Crous and Groenewald 2013

Apiospora xenocordella	CBS 595.66	KF144926	KF144971	–	–	Crous and Groenewald 2013
Apiospora yunnanum	**MFLUCC 15-0002**	KU940147	KU863135	–	–	Dai et al. 2016
Arthrinium austriacum	GZU 345006	MW208929	MW208860	–	–	Pintos and Alvarado 2021
Arthrinium caricicola	AP23518	MK014871	MK014838	MK017977	MK017948	Pintos et al. 2019
Arthrinium crenatum	**CBS 146353**	MW208931	MW208861	MW221923	MW221917	Pintos and Alvarado 2021
Arthrinium curvatum var. *curvatum*	AG191036	MW208935	MW208862	MW221924	–	Pintos and Alvarado 2021
Arthrinium curvatum var. *minus*	AP25418	MK014872	MK014839	MK017978	MK017949	Pintos et al. 2019
Arthrinium japonicum	IFO 31098	AB220264	AB220358	AB220311	–	Ogawa et al. 2005
Arthrinium luzulae	AP7619-3	MW208937	MW208863	MW221925	MW221919	Pintos and Alvarado 2021
Arthrinium morthieri	GZU 345043	MW208938	MW208864	MW221926	MW221920	Pintos and Alvarado 2021
Arthrinium sphaerospermum	AP25619	MW208943	MW208865	–	–	Pintos and Alvarado 2021
Arthrinium sporophleoides	GZU 345102	MW208944	MW208866	–	MW208866	Pintos and Alvarado 2021
Arthrinium sporophleum	AG19067	MW208945	–	MW221928	MW221921	Pintos and Alvarado 2021
Arthrinium trachycarpum	**CFCC 53038**	MK301098	–	MK303394	MK303396	Jiang et al. 2020
Arthrinium urticae	IMI 326344	AB220245	AB220339	AB220292	–	Ogawa et al. 2005
Nigrospora aurantiaca	**CGMCC 3.18130**	KX986064	KX986098	KY019465	KY019295	Wang et al. 2018
	LC7034	KX986093	–	KY019598	KY019394	Wang et al. 2018

Nigrospora bambusae	**CGMCC 3.18327**	KY385307	–	KY385319	KY385313	Dai et al. 2016
	LC7244	KY385306	–	KY385320	KY385314	Wang et al. 2017
Nigrospora camelliae-sinensis	**CGMCC 3.18125**	KX985986	KX986103	KY019460	KY019293	Wang et al. 2017
	LC4460	KX986015	–	KY019538	KY019353	Wang et al. 2017
Nigrospora chinensis	**CGMCC 3.18127**	KX986023	KX986107	KY019462	KY019422	Wang et al. 2017
	LC4593	KX986024	–	KY019546	KY019443	Wang et al. 2017
	MFLUCC 14-0109	MZ463152	MZ463193	–		This study
	MFLUCC 18-1215	MZ463150	MZ463191	–		This study
Nigrospora gorlenkoana	**CBS 480.73**	KX986048	KX986109	KY019456	KY019420	Wang et al. 2017
Nigrospora guilinensis	LC7301	KX986063	–	KY019608	KY019404	Wang et al. 2017
Nigrospora hainanensis	**CGMCC 3.18129**	KX986091	KX986112	KY019464	KY019415	Wang et al. 2017
	LC6979	KX986079	–	KY019586	KY019416	Wang et al. 2017
Nigrospora lacticolonia	**CGMCC 3.18123**	KX985978	KX986105	KY019458	KY019291	Wang et al. 2017
	LC7009	KX986087	–	KY019594	KY019454	Wang et al. 2017
Nigrospora musae	**CBS 319.34**	KX986076	KX986110	KY019455	KY019419	Wang et al. 2017
	LC6385	KX986042	–	KY019567	KY019371	Wang et al. 2017
Nigrospora oryzae	LC6759	KX986054	–	KY019572	KY019374	Wang et al. 2017
	LC6760	KX986055	–	KY019573	KY019375	Wang et al. 2017
Nigrospora osmanthi	**CGMCC 3.18126**	KX986010	KX986106	KY019461	KY019421	Wang et al. 2017
	LC4487	KX986017	–	KY019540	KY019438	Wang et al. 2017
Nigrospora pyriformis	**CGMCC 3.18122**	KX985940	KX986100	KY019457	KY019290	Wang et al. 2017
	LC2688	KX985941	–	KY019468	KY019297	Wang et al. 2017
Nigrospora rubi	**CGMCC 3.18326**	KX985948	KX986102	KY019475	KY019302	Wang et al. 2017
Nigrospora sphaerica	**LC13523**	MN215807	–	MN329970	MN264046	Wang et al. 2017

	LC7295	KX985933	–	KY019603	KY019398	Wang et al. 2017
	GZAC O37S13	MZ463153	–	–		
Nigrospora sp. 1	LC2725	KX985960	KX986104	KY019487	KY019313	Wang et al. 2017
Nigrospora sp. 1	LC4566	KX986022	–	KY019545	KY019354	Raza et al. 2019
Nigrospora sp. 2	LC6704	KX986047	KX986108	KY019571	KY019373	Wang et al. 2017
Nigrospora vesicularis	**CGMCC 3.18128**	KX986088	KX986099	KY019463	KY019294	Wang et al. 2017
	LC0322	KX985939	–	KY019467	KY019296	Wang et al. 2017
Nigrospora zimmermanii	**CBS 290.62**	KY385309	–	KY385317	KY385311	Wang et al. 2017
	CBS 984.69	KY385310	–	KY385322	KY385316	Wang et al. 2017
Pseudomassaria corni	MFLUCC 14-0544	KR092791	–	–	–	Wang et al. 2017
Pseudomassaria chondrospora	MFLUCC 14-0545	KR092790	KR092779	–	–	Wang et al. 2017
Pseudomassaria sepincoliformis	CBS 129022	JF440984	–	–	–	Wang et al. 2017

Table S2. Strains used for phylogenetic tree construction in related Xylariacea

**Species**	**Strains**	**GenBank Accession No.**				**Reference**
		**ITS**	**LSU**	**TUB2**	**RPB2**	
Alloanthostomella rubicola	MFLUCC 16-0479	KX533455	KX533456	KX789494	KX789493	Bitzer et al. 2008
Amphirosellinia fushanensis	**91111209 (HAST)**	NR153514	—	GQ495950	GQ848339	Hsieh et al. 2010
Amphirosellinia nigrospora	**91092308 (HAST)**	NR153513	—	GQ495951	GQ848340	Hsieh et al. 2010
Annulohypoxylon annulatum	**CBS 140775**	NR153579	KY610418	KX376353	KY624263	Sir et al. 2016
Annulohypoxylon areolatum	**MFLUCC 14-1233**	KX376327	—	KX376344	—	Kuhnert et al. 2017
Annulohypoxylon atroroseum	ATCC 76081	AF201712	KY610422	DQ840083	KY624233	Wendt et al. 2018

Annulohypoxylon bahnphadengense	STMA 13115	KX376338	—	KX376347	—	Kuhnert et al. 2017
Annulohypoxylon cf. *truncatum*	EKTX140201	KX376331	—	KX376351	—	Kuhnert et al. 2017
Annulohypoxylon elevatidiscus	**BCRC34014**	—	—	AY951656	—	Hsieh et al. 2005
Annulohypoxylon fulvum	**MUCL 54617**	KX376336	—	KX376355	—	Kuhnert et al. 2017
Annulohypoxylon ilanense	YMJ 37	—	—	AY951657	—	Hsieh et al. 2005
Annulohypoxylon leptascum	MFLUCC 13-0587	KU604576	—	KU604580	—	Sir et al. 2016
Annulohypoxylon massivum	**MUCL 47218**	AM749938	—	KC977276	—	Bitzer et al. 2008
Annulohypoxylon michelianum	CBS 119993	KX376320	KY610423	KX271239	KY624234	Wendt et al. 2018
Annulohypoxylon microdiscum	BCRC34018	EF026137	—	—	AY951660	Kuhnert et al. 2017
Annulohypoxylon moniliformis	**MFLUCC 18-1214**	MZ463121	—	MZ998970	—	This study
Annulohypoxylon moriforme	CBS 123579	KU683751	KY610425	KX271261	KU684279	U'Ren et al. 2016
Annulohypoxylon nitens	**MFLUCC 12-0823**	KJ934991	KJ934992	KJ934993	KJ934994	Daranagama et al. 2015
Annulohypoxylon nouraguense	**MUCL 54607**	KX376335	—	KX376348	—	Kuhnert et al. 2017
Annulohypoxylon purpureopigmentum	MUCL 54616	KC968942	—	KC977306	—	Kuhnert et al. 2014
Annulohypoxylon squamulosum	**BCRC34022**	EF026139	—	AY951665	—	Hsieh et al. 2005
Annulohypoxylon stygium	MUCL 54601	KY610409	KY610475	KX271263	KY624292	Wendt et al. 2018
Annulohypoxylon substygium	STMA 14066	KU604575	—	KU159526	—	Sir et al. 2016
Annulohypoxylon thailandicum	**MFLUCC 13-0118**	KP744434	KP744476	KX376349	—	Liu et al. 2015
Annulohypoxylon truncatum	**CBS 140778**	NR153580	KY610419	KX376352	KY624277	Wendt et al. 2018
	EKTX14006	KX376329	—	KX376352	—	Kuhnert et al. 2017

Annulohypoxylon urceolatum	YMJ 92090413	—	—	AY951670	—	Hsieh et al. 2005
Annulohypoxylon violaceopigmentum	**MFLUCC 14-1225**	KX376326	—	KX376343	—	Kuhnert et al. 2017
Annulohypoxylon viridistratum	**MFLUCC 14-1224**	KX376325	—	KX376342	—	Kuhnert et al. 2017
Annulohypoxylon yungensis	STMA 14049	KX376324	—	KX376341	—	Kuhnert et al. 2017
Anthocanalis sparti	**MFLUCC 14-0557**	KP297395	KP340537	KP406606	KP340523	Daranagama et al. 2015
Anthostomella eucalyptorum	CBS 120036	DQ890026	—	—	—	Crous et al. 2006
Anthostomella helicofissa	**MFLUCC 14-0173**	KP297406	—	KP406617	KP340534	Daranagama et al. 2015
Anthostomelloides krabiensis	**MFLUCC 15-0678**	KX305927	KX305928	—	KX305929	Tibpromma et al. 2017
Anthostomelloides leucospermi	**CBS 110126**	NR153510	—	—	—	Marincowitz et al. 2008
Astrocystis concavispora	**MFLUCC 14-0174**	KP297404	KP340545	KP406615	KP340532	Daranagama et al. 2015
Barrmaelia macrospora	BM	KC774566	—	MF489014	MF488995	Voglmayr et al. 2017
Biscogniauxia arima	**WSP 122**	EF026150	—	AY951672	GQ304736	Hsieh et al.2010
Biscogniauxia atropunctata	YMJ 128	JX507799	—	AY951673	JX507778	Hsieh et al. 2012
Biscogniauxia marginata	**MFLUCC 12-0740**	KJ958407	KJ958408	KJ958406	KJ958409	Daranagama et al. 2015
Biscogniauxia nummularia	**MUCL 51395**	KY610382	KY610427	KX271241	KY624236	Wendt et al. 2018
Biscogniauxia repanda	ATCC 62606	KY610383	KY610428	KX271242	KY624237	Wendt et al. 2018
Brunnelperidium gracilentum	**MFLUCC 14-0011**	KP297400	KP340542	KP406611	KP340528	Daranagama et al. 2015
Brunneiperidium involucratum	**MFLUCC 14-0009**	KP297399	KP340541	KP406610	KP340527	Daranagama et al. 2015
Camillea obularia	ATCC 28093	KY610384	KY610429	KX271243	KY624238	Wendt et al. 2018
Camillea tinctor	YMJ 363	JX507806	—	JX507795	JX507790	Wendt et al. 2018
Clypeosphaeria mamillana	**CBS 140735**	KT949897	MH554225	MH704637	MF489001	Jaklitsch et al. 2016
Collodiscula bambusae	**GZU H0102**	KP054279	KP054280	KP276674	KP276675	Li et al. 2015
Collodiscula fangjingshanensis	**GZUH0109**	KR002590	NG058915	KR002589	KR002592	Li et al. 2015

Collodiscula japonica	CBS 124266	JF440974	MH874889	KY624316	KY624273	Wendt et al. 2018
Daldinia andina	**CBS 114736**	KU684017	KY610430	KC977259	KY624239	Wendt et al. 2018
Daldinia bambusicola	**CBS 122872**	NR152464	KY610431	AY951688	KY624241	Wendt et al. 2018
	MFLUCC 11-0605	KU940155	KU863143	—	KU940181	Dai et al. 2016
Daldinia brachysperma	BCC 33676	MN153854	MN153871	MN172205	—	Wongkanoun et al. 2019
Daldinia caldariorum	MUCL 49211	AM749934	KY610433	KC977282	KY624242	Wendt et al. 2018
Daldinia chiangdaoensis	BCC 88221	MN153851	MN153868	MN172198	MN172209	Wongkanoun et al. 2019
	**BCC 88220**	MN153850	MN153867	MN172197	MN172208	Wongkanoun et al. 2019
Daldinia childiae	**CBS 122881**	MH863249	MH874773	KU684129	KU684290	U'Ren et al. 2016
Daldinia concentrica	CBS 113277	AY616683	KY610434	KC977274	KY624243	Wendt et al. 2018
Daldinia decipiens	**CBS 122879**	MH863247	MH874771	AY951694	—	Vu et al. 2019
Daldinia dennisii	**CBS 114741**	MH862968	MH874530	KC977262	KY624244	Wendt et al. 2018
Daldinia eschscholtzii	MUCL 45435	JX658484	KY610437	KC977266	KY624246	Wendt et al. 2018
Daldinia flavogranulata	BCC 89365	MN153857	MN153874	MN172201	MN172212	Wongkanoun et al. 2019
Daldinia flavogranulata	**BCC 89363**	MN153856	MN153873	MN172200	MN172211	Wongkanoun et al. 2019
Daldinia loculatoides	**CBS 113279**	MH862918	MH874491	KX271246	KY624247	Wendt et al. 2018
Daldinia macaronesica	**CBS 113040**	JX658504	KY610477	KX271266	KY624294	Wendt et al. 2018
Daldinia petriniae	**MUCL 49214**	AM749937	KY610439	KC977261	KY624248	Wendt et al. 2018
Daldinia phadaengensis	BCC 89350	MN153853	MN153870	MN172196	MN172207	Wongkanoun et al. 2019
	**BCC 89349**	MN153852	MN153869	MN172195	MN172206	Wongkanoun et al. 2019
Daldinia placentiformis	MUCL 47603	AM749921	KY610440	KC977278	KY624249	Wendt et al. 2018
Daldinia pyrenaica	MUCL 53969	KY610413	KY610413	KY624312	KY624274	Wendt et al. 2018
Daldinia singularis	YMJ 120	—	—	AY951700	—	Hsieh et al.2005
Daldinia steglichii	**MUCL 43512**	KY610399	KY610479	KX271269	KY624250	Wendt et al. 2018

Daldinia theissenii	**CBS 113044**	KY610388	KY610441	KX271247	KY624251	Wendt et al. 2018
Daldinia vernicosa	**CBS 119316**	NR152501	KY610442	KC977260	KY624252	Wendt et al. 2018
Diatrype disciformis	**MFLUCC 15-0538**	AJ390410	KR092784	—	—	Senanayake et al. 2015
Durotheca comedens	**YMJ 90071615**	EF026128	—	EF025613	JX507793	Ju et al. 2003
Durotheca crateriformis	**GMBC0205**	MH645426	MH645425	MH049441	MH645427	Long et al. 2019
Durotheca depressa	**BCC23016**	—	—	GQ160491	—	Læssøe et al. 2013
Durotheca eurima	**GMBC0060**	MH645419	MH645421	MH049437	MH645422	Long et al. 2019
Durotheca guizhouensis	GMBC0206	MH645429	MH645430	MH049446	MH645431	Long et al. 2019
Durotheca rogersii	**GMBC0204**	MH645433	MH645434	MH049449	MH645435	Long et al. 2019
Euepixylon sphaeriostomum	**JDR 261**	GU292821	—	GQ470224	GQ844774.1	Hsieh et al.2010
Entoleuca mammata	JDR 100	GU300072	—	GQ470230	GQ844782	Hsieh et al.2010
Entosordaria perfidiosa	BW3	MF488992	—	MF489020	MF489002	Voglmayr et al. 2017
Graphostroma platystoma	**CBS 270.87**	JX658535	DQ836906	HG934108	KY624296	Wendt et al. 2018
Hypocreodendron sanguineum	**JDR 169**	GU322433	—	GQ487710	GQ844819	Hsieh et al.2010
Hypomontagnella barbarensis	**STMA 14081**	MK131720	MK131718	MK135891	MK135893	Lambert et al. 2019
Hypomontagnella monticulosum	**MUCL 54604**	KY610404	KY610487	KX271273	KY624305	Wendt et al. 2018
Hypomontagnella submonticulosum	CBS 115280	JX658449	KY610457	KC977267	KY624226	Wendt et al. 2018
Hypoxylon addis	**MUCL 52797**	KC968931	—	KC977287	—	Kuhnert et al. 2017
Hypoxylon aeruginosum	**MUCL 54620**	KC968941	—	KC977305	—	Kuhnert et al. 2014
Hypoxylon argillaceum	CBS 527.63	KU683764	—	KU684187	KU684283	U'Ren et al. 2016
Hypoxylon aff. rubiginosum	**MUCL 57724**	MT214999	MT214994	MT212241	MT212237	Pourmoghaddam et al. 2020
Hypoxylon anthochroum	BCRC34050	JN660819	—	AY951703	—	Hsieh et al. 2005

Hypoxylon baihualingense	**H14**	MG490190	—	MH790276	—	Ma et al. 2018
Hypoxylon baruense	**UCH9545**	MN056428	—	MK908142.	—	Cedeño–Sanchez et al. 2019
Hypoxylon begae	BCRC 34051	JN660820	—	AY951704	—	Hsieh et al. 2005
Hypoxylon bellicolor	**UCH9543**	MN056425	—	—	MK908139	Cedeno-Sanchez et al. 2019
Hypoxylon brevisporum	BCRC33809	JN660821	—	AY951705	—	Hsieh et al.2005
Hypoxylon calileguense	**STMA 14059**	KU604566	—	KU604579	—	Sir et al. 2016
Hypoxylon carneum	MUCL 54177	KY610400	KY610480	KX271270	KY624297	Wendt et al. 2018
Hypoxylon cercidicola	CBS 119009	KU683766	KY610444	KU684189	KY624254	Wendt et al. 2018
Hypoxylon cf. *subticinense*	MUCL 53752	KC968913	—	KC977297	—	Kuhnert et al. 2014
Hypoxylon chionostomum	STMA 14060	KU604563	—	—	—	Sir et al. 2016
Hypoxylon cinnabarinum	YMJ 43	JN979409	—	AY951709	—	Hsieh et al.2005
Hypoxylon crocopeplum	CBS 119004	KC968907	KY610445	KC977268	KY624255	Wendt et al. 2018
Hypoxylon dieckmannii	BCRC34058	JN979413	—	AY951712	—	Hsieh et al.2005
Hypoxylon duranii	BCRC34060	JN979414	—	AY951714	—	Hsieh et al.2005
Hypoxylon endophyticum	**MFLUCC 18-1206**	MZ463115	MZ463160	MZ998938	MZ613967	This study
	MFLUCC 18-1209	MZ463134	MZ463177	MZ998953	MZ970698	This study
	MFLUCC 18-1211	MZ463118	MZ463163	MZ998941	MZ690490	This study
	**MFLUCC 18-1208**	MZ463131	MZ463174	MZ998950	MZ970703	This study
	MFLUCC 18-1210	MZ463137	MZ463180	MZ998956	MZ970705	This study
	MUCL 53759	KC968910	—	KC977296	—	Wendt et al. 2018
Hypoxylon fendleri	MUCL 54792	KF234421	KY610481	KF300547	KY624298	Wendt et al. 2018
Hypoxylon ferrugineum	CBS 141259	KX090079	—	KX090080	—	Friebes et al. 2016
Hypoxylon flavoargillaceum	STMA 14062	KU604577	—	KU159532	—	Sir et al. 2016

Hypoxylon fragiforme	**MUCL 51264**	KM186294	KM186295	KM186293	KM186296	Wendt et al. 2018
	YMJ 387	JN979419	—	AY951719	—	Hsieh et al. 2005
Hypoxylon fraxinophilum	**STMA 12011**	KC968938	—	KC977301	—	Kuhnert et al. 2014
Hypoxylon fulvosulphureum	**MFLUCC 13-0589**	KP401576	—	KP401584	—	Sir et al. 2016
Hypoxylon fuscopurpureum	BCRC34067	JN979421	—	AY951721	—	Hsieh et al. 2005
Hypoxylon fuscum	**CBS 113049**	KY610401	KY610482	KX271271	KY624299	Wendt et al. 2018
Hypoxylon gibriacense	**MUCL 52698**	KC968930	—	AY951739	—	Kuhnert et al. 2014
Hypoxylon griseobrunneum	**CBS 331.73**	KY610402	MH872399	KC977303	KY624300	Wendt et al. 2018
Hypoxylon guilanense	**MUCL 57726**	MT214997	MT214992	MT212239	MT212235	Pourmoghaddam et al. 2020
Hypoxylon haematostroma	MUCL 47600	AM749924	KY610447	KC977279	KY624257	Wendt et al. 2018
Hypoxylon hercoglossi	**MFLUCC 18-1207**	MZ463125	MZ463168	—		This study
Hypoxylon hinnuleum	**CBS 286.62**	MH858152	MH869746	MK287575	MK287562	Sir et al. 2019
Hypoxylon howeanum	MUCL 47599	AM749928	KY610448	KC977277	KY624258	Wendt et al. 2018
Hypoxylon hypomiltum	MUCL 51845	KY610403	KY610449	KX271249	KY624302	Wendt et al. 2018
Hypoxylon investiens	CBS 118185	FJ185308	KY610451	FJ185299	KY624260	Platas et al. 2009
	CBS 118183	FJ185307	KY610450	FJ185298	KY624259	Platas et al. 2009
	YMJ 89062905	JN979428	—	AY951730	—	Hsieh et al.2005
Hypoxylon isabellinum	**MUCL 53308**	KC968935	—	KC977295	—	Kuhnert et al. 2014
Hypoxylon jaklitschii	**CBS 138916**	KM610290	—	KM610304	—	Kuhnert et al. 2015
Hypoxylon jecorinum	YMJ 39	JN979429	—	AY951731	—	Hsieh et al.2005
Hypoxylon lateripigmentum	**MUCL 53304**	NR155156	KY610486	KC977290	KY624304	Kuhnert et al. 2014
Hypoxylon lenormandii	CBS 119003	KC968943	KY610452	KC977273	KY624261	Wendt et al. 2018
Hypoxylon lienhwacheense	MFLUCC 14-1231	KU604558	—	KU159522	—	Sir et al. 2016
Hypoxylon lilloi	**STMA 14142**	KU604574	—	KU159537	—	Sir et al. 2016

Hypoxylon liviae	**CBS 115282**	KC968922	—	KC977265	—	Kuhnert et al. 2017
Hypoxylon lividicolor	**BCRC34076**	JN979432	—	AY951734	—	Hsieh et al. 2005
Hypoxylon lividipigmentum	**BCRC34077**	JN979433	MG685777	AY951735	—	Sir et al. 2016
Hypoxylon macrosporum	YMJ 47	JN979434	—	AY951736	—	Hsieh et al. 2005
Hypoxylon moschaticum	**MFLUCC 15-1155**	MZ463149	—	MZ998968		This study
Hypoxylon munkii	MUCL 53315	KC968912	—	KC977294	—	Kuhnert et al. 2014
Hypoxylon musceum	MUCL 53765	KC968926	KY610488	KC977280	KY624306	Wendt et al. 2018
Hypoxylon notatum	YJM 250	JQ009305	—	AY951739	—	Hsieh et al. 2010
Hypoxylon ochraceum	**MUCL 54625**	NR155158	—	KC977300	KY624271	Wendt et al. 2018
Hypoxylon officinalis	MFLUCC 14-0078	MZ463109	MZ463156	MZ998933	—	This study
	**MFLUCC 14-0075**	MZ463108	MZ463155	MZ613966	—	This study
	MFLUCC 21-0060	MZ463123	—	MZ998943	MZ690492	This study
Hypoxylon olivaceopigmentum	**DSM 107924**	MK287530	MK287542	MK287568	MK287555	Sir et al. 2019
Hypoxylon papillatum	**ATCC 58729**	NR155153	KY610454	KC977258	KY624223	Wendt et al. 2018
Hypoxylon perforatum	CBS 115281	AM749935	KY610455	KX271250	KY624224	Wendt et al. 2018
Hypoxylon petriniae	**CBS 114746**	NR155185	KY610491	KX271274	KY624279	Wendt et al. 2018
Hypoxylon pilgerianum	YMJ 92042505	JQ009310	—	AY951744	KY624308	Hsieh et al. 2005
Hypoxylon polyporoideum	BCRC34088	JQ009311	—	AY951747	—	Hsieh et al. 2005
Hypoxylon polyporus	STMA 14090	KU604570	—	KU159530	—	Sir et al. 2016
Hypoxylon porphyreum	CBS 119022	KC968921	KY610456	KC977264	KY624225	Kuhnert et al. 2014
Hypoxylon pulicicidum	**MUCL 49879**	JX183075	—	JX183072	—	Bills et al. 2012
	MUCL 53764	JX183077	—	JX183073	—	Bills et al. 2012
	CBS 122622	NG066188	KY610492	JX183074	KY624280	Wendt et al. 2018
	GZAC O37L14	MZ463124	MZ463167	MZ998944	MZ690493	This study

Hypoxylon rickii	**MUCL 53309**	NR137115	KY610416	KC977288	KY624281	Wendt et al. 2018
Hypoxylon rubiginosum	**MUCL 52887**	NR155152	KY610469	KY624311	KY624266	Wendt et al. 2018
Hypoxylon rutilum	YMJ 181	—	—	AY951752	—	Hsieh et al. 2005
Hypoxylon samuelsii	**MUCL 51843**	NR137159	KY610466	KC977286	KY624269	Wendt et al. 2018
Hypoxylon shearii var. *minor*	**BCRC34093**	EF026142	—	AY951753	—	Hsieh et al. 2005
Hypoxylon spegazzinianum	**STMA 14082**	KU604573	—	KU604582	—	Sir et al. 2016
Hypoxylon sporistriatatunicum	**UCH9542**	MN056426	—	MK908140	—	Cedeno-Sanchez et al. 2019
Hypoxylon subgilvum	YMJ 88113007	JQ009314	—	AY951754	—	Hsieh et al. 2005
Hypoxylon sublenormandii	JF13026	KM610291	—	KM610303	—	Kuhnert et al. 2015
Hypoxylon subrutiloides	F202416	FJ185304	—	FJ185281	—	Platas et al. 2009
Hypoxylon texense	**DSM 107933**	MK287536	MK287548	MK287574	MK287561	Kuhnert et al. 2017
Hypoxylon ticinense	CBS 115271	JQ009317	KY610471	AY951757	KY610471	Wendt et al. 2018
Hypoxylon trugodes	**MUCL 54794**	KF234422	KY610493	KF300548	KY624282	Wendt et al. 2018
Hypoxylon ulmophilum	**YMJ 350**	JQ009320	—	AY951760		Hsieh et al. 2005
Hypoxylon vinosopulvinatum	**BCRC34101**	JQ009321	—	AY951761	—	Hsieh et al. 2005
Hypoxylon vogesiacum	CBS 115273	KY610417	KY610417	KX271275	KY624283	Wendt et al. 2018
Induratia alba	**MONT 620**	AF324336	—	—	—	Worapong et al. 2001
Induratia brasiliensis	**LGMF 1256**	KY924494	—	—	MF510171	Pena et al., (2019)
Induratia camphorae	**NFCCI 3236**	KC481681	—	—	—	Meshram et al. 2017
Induratia cinnamomi	**BCC 38842**	GQ848369	—	—	—	Suwannarach et al. 2010
Induratia coffeana	**COAD 1842**	KM514680	—	KM514681	—	Hongsanan et al. 2015
Induratia crispans	**MONT 2347**	EU195297	—	—	—	Suwannarach et al. 2010
Induratia equiseti	**JCM 18233**	JX089322	—	—	—	Suwannarach et al. 2013

Induratia darjeelingensis	**NFCCI 3095**	NFCCI 3095	—	—	—	Saxena et al. 2014
Induratia fengyangensis	ZJLQ024	HM034855	HM034861	HM034842	HM034851	Zhang et al. 2010
Induratia ghoomensis	**NFCCI 3234**	KF537625	—	—	—	Meshram et al. 2015
Induratia indica	**NFCCI 3235**	KF537626	—	—	—	Meshram et al. 2015
Induratia kashayum	**NFCCI 2947**	KC481680	—	—	—	Meshram et al. 2013
Induratia musae	**JCM 18230**	JX089323	—	—	—	Suwannarach et al. 2010
Induratia oryzae	**JCM 18231**	JX089321	—	—	—	Suwannarach et al. 2010
Induratia rosea	**MONT 2098**	AY034665	—	—	—	Worapong et al. 2002
Induratia sp.	MFLUCC 15-1218	MZ463110	—	—	—	This study
Induratia suthepensis	**JCM 18232**	JN558830	—	—	—	Suwannarach et al. 2010
Induratia sutura	**MSUB 2380**	JF938595	—	—	—	Kudalkar et al. 2012
Induratia thailandica	**MFLUCC 17-2669**	MK762707	MK762714	MK791283	MK776960	Samarakoon et al. 2020
Induratia tigerensis	**NFCCI 3172**	JQ409998	—	—	—	Saxena et al. 2015
Induratia vitigena	**MONT P-15**	AY100022	—	—	—	Daisy et al. 2002
Induratia yucatanensis	**MEXU 25511**	FJ917287	—	—	—	González et al. 2009
Induratia yunnanensis	**CGMCC 3.18908**	MG866046	MG866038	MG866059	MG866066	Chen et al. 2019
Induratia ziziphi	**MFLUCC 17-2662**	MK762705	MK762712	MK791281	MK776958	Samarakoon et al. 2020
Jackrogersella cohaerens	CBS 119126	KC477233	KY610497	KY624314	KY624270	Wendt et al. 2018
Jackrogersella minutella	CBS 119015	KY610381	KY610424	KX271240	KY624235	Wendt et al. 2018
Jackrogersella multiformis	**CBS 119016**	NR154784	KT281893	KX271262	KY624290	Wendt et al. 2018
Kretzschmaria clavus	YMJ 114	EF026126	—	EF025611	GQ844789	Hsieh et al. 2010
Kretzschmaria deusta	CBS 163.93	KC477237	KY610458	KX271251	KY624227	Wendt et al. 2018
Kretzschmaria guyanensis	89062903 (HAST)	GU300079	—	GQ478214	GQ844792	Hsieh et al. 2010
Kretzschmaria lucidula	YMJ 112	EF026125	—	EF025610	GQ844790	Hsieh et al. 2010

Kretzschmaria neocaledonica	94031003 (HAST)	GU300078	—	GQ478213	GQ844788	Hsieh et al. 2010
Kretzschmaria pavimentosa	YMJ 109	GU300077	—	GQ478212	GQ844787	Hsieh et al. 2010
Kretzschmaria sandvicensis	YMJ 113	GU300076	—	GQ478211	GQ844786	Hsieh et al. 2010
Kretzschmariella culmorum	JDR 88	KX430043	—	KX430046	KX430045	Fournier et al. 2018
Lopadostoma gastrinum	**CBS 134632**	NR132030	MH877560	—	—	Vu et al. 2019
Lopadostoma insulare	**CBS 133214**	NR132031	—	—	—	Jaklitsch et al. 2014
Lunatiannulus irregularis	**MFLUCC 14-0014**	KP297398	KP340540	KP406609	KP340526	Daranagama et al. 2015
Natonodosa speciosa	**CLM-RV86**	MF380435	MF380435	—	MH745150	Heredia et al. 2020
Nemania abortiva	**BISH467**	GU292816	—	GQ470219	KU684251	Hsieh et al.2010
Nemania aenea	CBS 680.86	AJ390427	—	—	—	Sanchez-Ballesteros et al. 2000
Nemania aenea var. *aureolutea*	ATCC 60819	AF201704	—	—	—	Pinto-Sherer et al. 2000
Nemania beaumontii	405 (HAST)	GU292819	—	GQ470222	GQ844772	Hsieh et al. 2010
Nemania bipapillata	5336	JQ862691	JQ862649	JX868546	—	Chen et al. 2013
	90080610 (HAST)	GU292818	—	GQ470221	GQ844771	Hsieh et al. 2010
Nemania chestersii	ATCC 38988	AJ390430	—	DQ631949	—	Sanchez-Ballesteros et al. 2000
Nemania dendrobii	**MFLUCC 18-1213**	MZ463137	MZ463181	MZ998957	MZ970708	This study
	**MFLUCC 18-1212**	MZ463135	MZ463178	MZ998954	MZ970699	This study
Nemania diffusa	**91020401 (HAST)**	GU292817	—	GQ470220	GQ844769	Hsieh et al. 2010
	JZB3370003	MT509577	—	—	MT512901	Manawasinghe et al. 2021
	MFLUCC 14-0139	MZ463143	MZ463185	MZ998962	—	This study
Nemania illita	YMJ 236	EF026122	—	EF025608	—	Hsieh et al. 2010
Nemania macrocarpa	**CBS 109567**	MH862830	MH874423	GQ470226	GQ844776	Vu et al. 2019

Nemania maritima	**89120401 (HAST)**	GU292822	—	GQ470225	GQ844775	Hsieh et al. 2010
Nemania orchidacearum	**MFLUCC 14-0138**	MZ463141	MZ463183	MZ998960	MZ970706	This study
	**MFLUCC 14-0105**	MZ463114	MZ463159	MZ998937	—	This study
Nemania plumbea	JF TH-04-01	DQ641634	—	DQ631952	—	Tang et al. 2007
Nemania primolutea	**91102001 (HAST)**	EF026121	—	EF025607	GQ844767	Tang et al. 2007
Nemania serpens	ATCC 16078	AJ390431	—	—	—	Sanchez-Ballesteros et al. 2000
Nemania serpens var. *macrospora*	ATCC 60822	AJ390433	—	—	—	Sanchez-Ballesteros et al. 2000
Neoxylaria arengae	**MFLUCC 15-0292**	MT496747	—	—	MF459058	Konta et al. 2020
Neoxylaria juruensis	92042501 (HAST)	GU322439	—	GQ495932	GQ844825	Konta et al. 2020
Obolarina dryophila	MUCL 49882	GQ428316	GQ428316	GQ428322	KY624284	Wendt et al. 2018
Podosordaria mexicana	WSP176	GU324762	—	GQ844840	GQ853039	Hsieh et al. 2010
Podosordaria muli	**WSP167**	GU324761	—	GQ844839	GQ853038	Hsieh et al. 2010
Poronia pileiformis	**WSP 88113001**	GU324760	—	GQ502720	GQ853037	Hsieh et al. 2010
Poronia punctata	**CBS 656.78**	KT281904	KY610496	KX271281	KY624278	Wendt et al. 2018
Pyrenopolyporus hunteri	**MUCL 52673**	KY610421	KY610472	KU159530	KY624309	Wendt et al. 2018
Pyrenopolyporus laminosus	**MUCL 53305**	NR154296	KY610485	KC977292	KY624303	Wendt et al. 2018
Pyrenopolyporus nicaraguensis	CBS 117739	AM749922	KY610489	AM749922	KY624307	Wendt et al. 2018
Rhopalostroma angolense	CBS 126414	KY610420	KY610459	KX271277	KY624228	Wendt et al. 2018
Rosellinia aquila	MUCL 51703	KY610392	KY610460	KX271253	KY624285	Wendt et al. 2018
Rosellinia buxi	JDR 99	GU300070	—	GQ470228	GQ844780	Hsieh et al.2010
Rosellinia corticium	MUCL 51693	KY610393	KY610461	KX271254	KY624229	Wendt et al. 2018
Rosellinia lamprostoma	YMJ 89112602	EF026118	—	EF025604	GQ844778	Hsieh et al.2010
Rosellinia necatrix	CBS 349.36	AY909001	KF719204	KY624310	—	Vu et al. 2019

Rosellinia sanctae-cruciana	90072903(HAST)	GU292824	—	GQ470227	GQ844777	Hsieh et al.2010
Rostrohypoxylon terebratum	**CBS 119137**	NR137677	NG057759	DQ840097	DQ631954	Wendt et al. 2018
Ruwenzoria pseudoannulata	**MUCL 51394**	NR137733	NG059787	KX271278	KY624286	Stadler et al. 2009
Sarcoxylon compunctum	CBS 359.61	KT281903	KY610462	KX271255	KY624230	Wendt et al. 2018
Stilbohypoxylon elaeicola	YMJ 173	EF026148	—	EF025616	GQ844826	Hsieh et al.2010
Stilbohypoxylon quisquiliarum	YMJ 172	EF026119	—	EF025605	GQ853020	Hsieh et al.2010
Thamnomyces dendroidea	**CBS 123578**	NR154472	KY610467	KY624313	KY624232	Stadler et al. 2010
Xylaria acuminatilongissima	**95060506 (HAST)**	EU178738	—	GQ502711	GQ853028	Ju and Hsieh 2007
Xylaria acuta	AFTOL-ID 63	DQ491493	AY544676	—	DQ247797	Lutzoni et al. 2004
Xylaria adscendens	570 (HAST)	GU300101	—	GQ487708	GQ844817	Hsieh et al.2010
Xylaria aethiopica	YMJ 1136	MH790445	—	MH785221	MH785222	Fournier et al. 2018
Xylaria allantoidea	94042903 (HAST)	GU324743	—	GQ502692	GQ848356	Hsieh et al.2010
Xylaria amphithele	529 (HAST)	GU300083	—	GQ478218	GQ844796	Hsieh et al.2010
Xylaria apiculata	EF6	MF927538	—	—	—	Poornima et al. 2017
Xylaria aphylli	**MFLUCC 21-0059**	MZ463116	MZ463161	MZ998939	MZ613968	This study
Xylaria apoda	90080804 (HAST)	GU322437	—	GQ495930	GQ844823.	Hsieh et al.2010
Xylaria arbuscula	CBS 126415	KY610394	KY610463	KX271257	KY624287	Wendt et al. 2018
	CBS 126416	MH864102	MH875561	—	—	Vu et al. 2019
Xylaria arbuscula var. *plenofissura*	93082814 (HAST)	GU339495	—	GQ478225	GQ844804	Hsieh et al.2010
Xylaria areolata	543 (HAST)	GU300080	—	GQ478215	GQ844793	Hsieh et al.2010
Xylaria atrodivaricata	**95052001 (HAST)**	EU178739	—	GQ502713	GQ853030	Ju and Hsieh 2007
Xylaria atrosphaerica	91111214 (HAST)	GU322459	—	GQ495953	GQ848342	Hsieh et al.2010
Xylaria badia	5256	JQ862687	JQ862643	JX868543	—	Chen et al. 2013

Xylaria bambusicola	MFLUCC 11-0606	KU940160	KU863148	—	KU940183	Dai et al. 2016
	**WSP 205**	NR153200	—	AY951762	GQ844802	Hsieh et al.2010
Xylaria berteri	90112623 (HAST)	GU324749	—	—	—	Hsieh et al.2010
	JDR 256	GU324750	—	GQ502698	GQ848363	Hsieh et al.2010
	YMJ 95101511	KC473562	—	KC473561	—	Fu et al. 2013
	MFLUCC 14-0095	MZ463112	MZ463157	MZ998935	MZ490538	This study
	MFLUCC 14-0102	MZ463113	MZ463158	MZ998936	MZ490539	This study
	MFLUCC 14-0110	MZ463111	—	MZ998934	—	This study
	MFLUCC 14-0117	MZ463130	MZ463173	MZ998949	—	This study
	MFLUCC 14-0126	MZ463128	MZ463171	MZ998947	MZ970695	This study
	MFLUCC 14-0143	MZ463144	MZ463186	MZ998963	—	This study
	MFLUCC 14-0150	MZ463147	MZ463189	MZ998966	MZ970707	This study
	MFLUCC 14-0158	MZ463146	MZ463188	MZ998965	—	This study
	MFLUCC 21-0061	MZ463127	MZ463170	MZ998946	—	This study
Xylaria brunneovinosa	**720 (HAST)**	NR153201	—	GQ502706	GQ853023	Hsieh et al.2010
Xylaria castorea	**ATCC 76020**	AF163030	—	—	—	Lee et al. 2000
	PDD 600	GU324751	—	GQ502703	GQ853018	Hsieh et al.2010
Xylaria cf. *castorea*	91092303 (HAST)	GU324752	—	GQ502704	GQ853019	Hsieh et al.2010
Xylaria cf. *heliscus*	88113010 (HAST)	GU324742	—	GQ502691	GQ848355	Hsieh et al.2010
Xylaria chaiyaphumensis	**SWUF17-49.2**	MT622775	—	—	—	Wangsawat et al. 2021
Xylaria chrysanthi	**MFLUCC 21-0014**	MZ463133	MZ463176	MZ998952	MZ970697	This study
Xylaria cirrata	**664 (HAST)**	EU179863	—	GQ502707	GQ853024	Hsieh et al.2010
Xylaria coccophora	786 (HAST)	GU300093	—	GQ487701	GQ844809	Hsieh et al.2010
Xylaria cranioides	226 (HAST)	GU300075	—	GQ478210	GQ844785	Ju and Hsieh 2007

Xylaria crozonensis	398 (HAST)	GU324748	—	GQ502697	GQ848361	Hsieh et al.2010
Xylaria cubensis	GENT 159	—	—	GQ502702	GQ853017	Hsieh et al.2010
	477 (HAST)	—	—	GQ502699	GQ848364	Hsieh et al.2010
	JDR 860	GU991523	—	GQ502700	GQ848365	Hsieh et al.2010
Xylaria culleniae	JDR 189	GU322442	—	GQ495935	GQ844829	Hsieh et al.2010
Xylaria curta	494 (HAST)	GU322444	—	GQ495937	GQ844831	Hsieh et al.2010
	92092022 (HAST)	GU322443	—	GQ495936	GQ844830	Hsieh et al.2010
Xylaria cf. *curta*	GZAC O36L23	MZ463122	MZ463166	MZ998942	MZ690491	This study
Xylaria cf. *glebusa*	431 (HAST)	GU322462	—	GQ495956	GQ848345	Hsieh et al.2010
Xylaria dendrobii	**MFLUCC 14-0137**	MZ463140	—	MZ998959	—	This study
	GZAC O6LA2	MZ463148	MZ463190	MZ998967	—	This study
Xylaria digitata	919 (HAST)	GU322456	—	GQ495949	GQ848338	Hsieh et al.2010
Xylaria discolor	**YMJ 1280**	JQ087405	—	JQ087414	JQ087411	Ju et al. 2012
Xylaria enterogena	785 (HAST)	GU324736	—	GQ502685	GQ848349	Hsieh et al.2010
Xylaria escharoidea	**658 (HAST)**	EU179864	—	GQ502709	GQ853026	Hsieh et al.2010
Xylaria feejeensis	JDR 180	GU322453	—	GQ495946	GQ848335	Hsieh et al.2010
	GZAC O30S21	MZ463117	MZ463162	MZ998940	MZ690489	This study
Xylaria fimbriata	491 (HAST)	GU324753	—	GQ502705	GQ853022	Hsieh et al.2010
Xylaria fissilis	367 (HAST)	GU300073		GQ470231	GQ844783	Hsieh et al.2010
Xylaria frustulosa	92092010 (HAST)	GU322451	—	GQ495944	GQ844838	Hsieh et al.2010
Xylaria globosa	775 (HAST)	GU324735	—	GQ502684	GQ848348	Hsieh et al.2010
Xylaria grammica	5228	JQ862677	JQ862638	JX868538	—	Chen et al. 2013
	479 (HAST)	GU300097	—	GQ487704	GQ844813	Hsieh et al.2010
	5151	JQ862665	JQ862626	JX868535	—	Chen et al. 2013

	BCC1002	AB625411	AB376679	—	—	Okane et al. 2012
	MFLUCC 14-0093	MZ463107	MZ463154	MZ613965	—	This study
	MFLUCC 14-0146	MZ463139	MZ463182	MZ998958	MZ970700	This study
Hypoxylon griseobrunneum	**CBS 331.73**	KY610402	MH872399	KC977303	KY624300	Wendt et al. 2018
Xylaria haemorrhoidalis	89041207 (HAST)	GU322464	—	GQ502683	GQ848347	Hsieh et al.2010
Xylaria hongkongensis	**GDGM 40058**	NR154905	—	—	—	Tang et al. 2013
	GZAC O32S24	MZ463119	MZ463164	—	MZ970702	This study
Xylaria hypoxylon	**CBS 122620**	AM993141	KY610495	KX271279	KY624231	Persoh et al. 2009
Xylaria ianthinovelutina	553 (HAST)	GU322441		GQ495934	GQ844828	Hsieh et al.2010
Xylaria insolita	YMJ 99090301-1251	MN655979	—	MN656983	MN656981	Hsieh et al. 2020
Xylaria intracolorata	90080402 (HAST)	GU324741	—	GQ502690	GQ848354	Hsieh et al.2010
Xylaria intraflava	725 (HAST)	EU179866	—	GQ502718	GQ853035	Ju and Hsieh 2007
Xylaria ischnostroma	SWUF18-22.1	MT622788	—	MW459244	—	Wangsawat et al. 2021
Xylaria karyophthora	DRH059	KY564220	—	—	KY564216	Husbands et al. 2017
Xylaria laevis	95072910 (HAST)	GU324747	—	GQ502696	GQ848360	Hsieh et al.2010
	419 (HAST)	GU324746	—	GQ502695	GQ848359	Hsieh et al.2010
	GZAC O33L12	MZ463120	MZ463165	MZ998969		This study
Xylaria liquidambaris	93090701 (HAST)	GU300094	—	GQ487702	GQ844810	Hsieh et al.2010
Xylaria longipes	**CBS 148.73**	KU683768	—	KU684204	KU684280	Vu et al. 2019
Xylaria longissima	**IRAN 2268C**	NR147567	—	—	—	Hashemi et al. 2015
Xylaria luteostromata	508 (HAST)	GU324739	—	GQ502688	GQ848352	Hsieh et al.2010
Xylaria mali	CBS 385.35	AF163040	MH867225	KU684205	KU684286	Hsieh et al.2010
Xylaria meliacearum	JDR 148	GU300084	—	GQ478219	GQ844797	Hsieh et al.2010
Xylaria melitensis	CS1223	MW513723	MW51376	—	—	Fournier et al. 2021

Xylaria microceras	414 (HAST)	GU300086	—	GQ478221	GQ844799	Hsieh et al.2010
Xylaria minima	**SWUF18-3.2**	MT622789	—	MW459245	—	Wangsawat et al. 2021
Xylaria montagnei	495 (HAST)	GU322455	—	GQ495948	GQ848337	Hsieh et al.2010
Xylaria multiplex	580 (HAST)	GU300098	—	GQ487705	GQ844814	Hsieh et al.2010
Xylaria muscula	520 (HAST)	GU300087	—	GQ478222	GQ844800	Hsieh et al.2010
Xylaria necrophora	DMCC3829	MT808616	—	MT812966	MT812957	Garcia-Aroca et al. 2021
Xylaria nigripes	653 (HAST)	GU324755	—	GQ502710	GQ853027	Hsieh et al.2010
Xylaria ochraceostroma	**401(HAST)**	EU179869	—	GQ502717	GQ853034	Ju and Hsieh 2007
Xylaria oligotoma	784 (HAST)	GU300092	—	GQ487700	GQ844808	Hsieh et al.2010
Xylaria ophiopoda	93082805 (HAST)	GU322461	—	GQ495955	GQ848344	Hsieh et al.2010
Xylaria oxyacanthae	JDR 859	GU322434	—	GQ495927	GQ844820	Hsieh et al.2010
Xylaria palmicola	604 (PDD)	GU322436	—	GQ495929	GQ844822	Hsieh et al.2010
Xylaria papulis (=*Xylaria hongkongensis*)	5118	JX868517	JQ862616	JX868529	—	Chen et al. 2013
Xylaria phyllocharis	528 (HAST)	GU322445	—	GQ495938	GQ844832	Hsieh et al.2010
Xylaria plebeja	91122401 (HAST)	GU324740	—	GQ502689	GQ848353	Hsieh et al.2010
Xylaria polymorpha	MUCL 49884	KY610408	KY610464	KX271280	KY624288	Wendt et al. 2018
Xylaria reevesiae	HMH-2010g	GU322435	—	GQ495928	GQ844821	Hsieh et al.2010
Xylaria regalis	920 (HAST)	GU324745	—	GQ502694	GQ848358	Hsieh et al.2010
X. reinkingii var.*microspora*	**SWUF17-19.1**	MT622769	—	MW459234	—	Wangsawat et al. 2021
Xylaria ripicola	**KH KA11-0060-1**	NR153251	—	—	KU554696	Kim et al. 2016
Xylaria schweinitzii	92092023 (HAST)	GU322463	—	GQ495957	GQ848346	Hsieh et al.2010
Xylaria scuposa	497 (HAST)	GU322458	—	GQ495952	GQ848341	Hsieh et al.2010
Xylaria sicula f. *major*	90071613 (HAST)	GU300081	MH869355	GQ478216	GQ844794	Vu et al. 2019

Xylaria sihanonthii	**SWUF18-1.3**	MT622785	—	MW459242	—	Wangsawat et al. 2021
Xylaria spinulosa	**GZUCC13016**	—	—	KM236099	KM236098	Li et al.2017
Xylaria striata	304 (HAST)	GU300089	—	GQ478224	GQ844803	Hsieh et al.2010
Xylaria subescharoidea	**YMJ 99060401-1188**	MN655980	—	MN656984	MN656982	Hsieh et al.2020
Xylaria subintraflava	**SWUF16-4.3**	MT622762	—	MW459230	—	Wangsawat et al. 2021
Xylaria subtropicalis	**X1**	MG013556	MG013566	—	MG013547	Chacón et al. 2019
Xylaria telfairii	421 (HAST)	GU324737	—	GQ502686	GQ848350	Hsieh et al.2010
Xylaria tentaculata	KA13-1325	KM077164	—	—	—	Kim et al. 2016
Xylaria thienhirunae	**SWUF17-44.1**	MT622771	—	—	—	Wangsawat et al. 2021
Xylaria tuberoides	475 (HAST)	GU300074	—	GQ478209	GQ844784	Hsieh et al.2010
Xylaria venosula	94080508 (HAST)	EF026149	—	EF025617	GQ844806	Ju et al.2007
	MFLUCC 14-0114	MZ463129	MZ463172	MZ998948	—	This study
	MFLUCC 21-0013	MZ463126	MZ463169	MZ998945	MZ970694	This study
	MFLUCC 21-0015	MZ463136	MZ463179	MZ998955	MZ970704	This study
	MFLUCC 21-0016	MZ463132	MZ463175	MZ998951	MZ970696	This study
	MFLUCC 21-0017	MZ463145	MZ463187	MZ998964	MZ970701	This study
Xylaria venustula	88113002 (HAST)	GU300091	—	GQ487699	GQ844807	Hsieh et al.2010
Xylaria vinacea	**SWUF18-2.1**	MT622781	—	MW459239	—	Wangsawat et al. 2021
Xylaria vivantii	HMH-2010h	GU322438	—	GQ495931	GQ844824	Hsieh et al.2010
Xylariaceae sp.	MFLUCC 14-0141	MZ463142	MZ463184	MZ998961	—	This study
